# Non-Targeted Effects Models Predict Significantly Higher Mars Mission Cancer Risk than Targeted Effects Models

**DOI:** 10.1038/s41598-017-02087-3

**Published:** 2017-05-12

**Authors:** Francis A. Cucinotta, Eliedonna Cacao

**Affiliations:** University of Nevada Las Vegas, Las Vegas, NV 89195 USA

## Abstract

Cancer risk is an important concern for galactic cosmic ray (GCR) exposures, which consist of a wide-energy range of protons, heavy ions and secondary radiation produced in shielding and tissues. Relative biological effectiveness (RBE) factors for surrogate cancer endpoints in cell culture models and tumor induction in mice vary considerable, including significant variations for different tissues and mouse strains. Many studies suggest non-targeted effects (NTE) occur for low doses of high linear energy transfer (LET) radiation, leading to deviation from the linear dose response model used in radiation protection. Using the mouse Harderian gland tumor experiment, the only extensive data-set for dose response modelling with a variety of particle types (>4), for the first-time a particle track structure model of tumor prevalence is used to investigate the effects of NTEs in predictions of chronic GCR exposure risk. The NTE model led to a predicted risk 2-fold higher compared to a targeted effects model. The scarcity of data with animal models for tissues that dominate human radiation cancer risk, including lung, colon, breast, liver, and stomach, suggest that studies of NTEs in other tissues are urgently needed prior to long-term space missions outside the protection of the Earth’s geomagnetic sphere.

## Introduction

The health risks from galactic cosmic ray (GCR) exposure to astronauts include cancer^1^, central nervous system effects^2, 3^, cataracts^4, 5^, circulatory diseases^3, 6, 7^ and acute radiation syndromes^5^. Cancer and cataracts are the main concern for space missions in low Earth orbit^8^, while for long-term space missions outside the protection of the Earth’s magnetic field cancer risks are predicted to exceed acceptable risk levels^9^, and non-cancer risks are of concern^1–3, 6, 7^ for the higher organ doses of long-term space missions compared to missions in low Earth orbit. Annual GCR organ absorbed doses and dose equivalents vary over the solar cycle between 0.1 and 0.2 Gy/y and 0.3 and 0.6 Sv/yr, respectively for average spacecraft shielding thicknesses^1, 8, 9^. Protons dominate absorbed doses, while heavy ions, low energy protons and helium particles, and neutrons make important contributions to dose equivalent because of their large quality factors. The high energies of GCR limit practical shielding amounts from providing significant attenuation^9^. The exploration of Mars will require missions of 900 days or longer with more than one year in deep space where exposures to all energies of GCR are unavoidable and doses only modestly decreased by radiation shielding.

There are several uncertainties in estimating space radiation cancer risks, including radiation quality and dose-rate effects, the transfer of epidemiology data between populations, and statistical and bias errors in epidemiology data. Of these uncertainties, estimating radiation quality effects due to the lack of human data for the heavy ions and other high LET radiations that occur in space, is the largest uncertainty^9, 10^. Both quantitative and qualitative differences between high and low LET cancer risks are possible with the later suggesting inadequacies in the use of relative biological effectiveness (RBE) factors as a basis for risk estimates. Experimental studies of tumor induction in mice and surrogate cancer risk endpoints in cell culture models are used to estimate radiation quality factors and their uncertainties^1^. In studies of tumor induction with heavy ions, Harderian gland tumors in female B6CF1 mice has been the only study that has used a sufficient number of particle types (>4) and low doses (<0.2 Gy) to make a detailed study of radiation quality effects^11–14^.

Energy deposition by particles in biomolecules, cells, and tissues depends on several physical aspects of particle tracks including a core of very high energy deposition events near the particles path, and a so-called penumbra of energetic electrons denoted as δ-rays liberated in ionization by primary particles and electrons, which may extend for 100’s of microns from the particles path^9^. Other considerations are the varying ranges or cells traversed in tissue of particles of similar linear energy (LET) but different charge number or kinetic energy, and the effects of projectile and target fragmentation caused by nuclear interactions^9^. Both theoretical analysis and experimental studies suggest that biological effectiveness is dependent on particle energy and charge number and not LET alone due to the above noted physical characteristics.

Non-targeted effects (NTEs) include bystander effects where cells traversed by heavy ions transmit oncogenic signals to nearby cells, and genomic instability in the progeny of irradiated cells^15–17^. Analysis of the Harderian gland studies^9, 14, 18^ and experiments for chromosomal aberrations at low dose^19, 20^ suggests a model based on NTEs is favored over a targeted effects (TE) model, where the later assumes a linear dose response model consistent with DNA damage and misrepair assumptions, while the former suggests a supra-linear dose response occurs which increases the risk at low doses compared to the TE model. The NTE model is supported by many mechanistic studies^15–17^ using micro-beams to direct radiation to targeted cells, medium transfer from irradiated to unirradiated cells, and inhibitors of reactive oxygen species, gap junctions and signaling pathways. The totality of these studies provide important evidence for NTEs, which should be prioritized in light of the scarcity of both human epidemiology and animal carcinogenesis studies for high LET radiation at the low doses and dose-rates that occur in space.

In this paper we considered a track structure model of tumor prevalence (TP) with TE and NTE assumptions, which is used to describe the Harderian gland experiment and extrapolate the results to make predictions for low dose and dose-rate GCR exposures. Results are compared to a recently published study using a LET dependent model fit to the same data^14^, and then applied to predictions of GCR exposures. In the remainder of this paper, we first discuss a Hazard function for TP using a track structure model consistent with either TE and NTE models. In the results section we first compare the model to experimental data for protons and heavy ions. We then use models of space radiation environments and radiation transport to compare the track structure model to the LET dependent model for nominal spacecraft shielding, and of the TE to NTE predictions.

## Methods

### Hazard Function for Tumor Prevalence

Tumor prevalence (TP) is described by a Hazard function, *H*, which is dependent on radiation type for γ-rays or particles with charge number *Z* and kinetic energy per atomic mass unit (u), *E* and fluence, *F* ^21^:

1 $$TP=1-{e}^{-H(Z,E,F)}$$

In experiments^11–14^ the HG tumor prevalence was expressed as the number of animals with tumor(s) divided by the total number of animals in the experimental group. In these publications, although there are two HG per mouse, one near each eye, however the number of animals with HG tumors was used as the numerator for the prevalence calculation.

Time-dependent factors in the Hazard function were described previously^21, 22^, however are suppressed herein because the experiments considered are restricted to tumor prevalence at an age of 600 d (irradiated 120 d old mice and held 16 for months = 120+480 = 600 d). For γ-rays we used the following form for the Hazard function

2 $${H}_{\gamma }={H}_{0}+[{\alpha }_{\gamma }D+{\beta }_{\gamma }{D}^{2}]S(D)$$

In Equation (2), *H*_*0*_ represents the background prevalence when animals are sacrificed, α_γ_ and β_γ_ are the linear and quadratic with dose induction terms, and *S*(*D*) the cell survival probability.

For considering particles using LET (*L*, in keV/μm), absorbed dose (*D*, in Gy) and fluence (*F*, in μm^−2^) while ignoring the contributions from δ-rays in cells not traversed by particles^23^, the mean number of particle hits per cell nucleus (*N*_*H*_) is given by:

3 $$F=\frac{6.24D}{L}$$

4 $${N}_{H}=\frac{6.24DA}{L}=F{A}_{nuc}$$

The diameter of the epithelial cell nucleus believed to be transformed into mouse HG tumors is 5.5 µm^11–14^, which corresponds to a cross-sectional cell nucleus area, *A*_*nuc*_ of about 24 µm^2^.

For particles, we considered a track structure model that extrapolates to low doses (or low fluence) to the functional form used in the NASA cancer risk assessment model^1, 20^, which is consistent with a TE model that assumed a linear dose response at low doses. We also considered a NTE model, which assumed a non-linear type response in addition to the linear dose term at low doses. We used these models to make a global fit to the data^12–14^. The Hazard function for particles is modified by the cell survival probability, *S*, and in the TE model given by:

5 $$H{}_{TE}(Z,E,F)=[{H}_{0}+{\rm{\Sigma }}F+\beta {D}^{2}]S:\,{\rm{TE}}\,{\rm{model}}$$

while in the NTE model it is given by:

6 $${H}_{NTE}(Z,E,F)=[{H}_{0}+{\rm{\Sigma }}F+\beta {D}^{2}+\eta ]S:\,{\rm{NTE}}\,{\rm{model}}$$

where Σ is denoted as a pseudo-biological action cross section for tumor induction in units of µm^2^ with the designation as “pseudo” given because time-dependent factors have been suppressed, which impact values for the cross-sectional area predicted by fits to the experiments. Also appearing in Equation (6) is the η function that represents the NTE contribution, which is parameterized as a function of LET, *L* by:

7 $$\eta ={\eta }_{0}\,L{e}^{-{\eta }_{1}L}[1-{e}^{-{N}_{Bys}}]$$

In Equation (7) *N*_*Bys*_ is evaluated in a manner similar to Equation (4), however using an area, *A*_*Bys*_, corresponding the number of bystander cells surrounding a cell traversed directly from a HZE particle that receive an oncogenic signal. The second factor on the right hand side of Equation (7) describes the “turning on” of NTE at very low doses, which is estimated at about 1 mGy from alpha-particle experiments^24–26^. The HG experiments do not provide data to determine at which dose or fluence level this occurs, and if it depends on radiation quality or the temporal patterns of irradiation.

Following the parametric track structure model described in prior reports^1, 20^ we write an effective pseudo-biological action cross section for TE per particle as:

8 $${\rm{\Sigma }}(Z,E)={{\rm{\Sigma }}}_{0}P(Z,E)+\frac{{\alpha }_{\gamma }\,L}{6.24}[1-P(Z,E)]\,P(Z,E)={[1-{e}^{(-\frac{{Z}^{\ast 2}}{\kappa {{v}_{c}}^{2}})}]}^{m}$$

where Z^*^ is the effective charge number of the particle, and *v*_*c*_ is the particle velocity relative to the velocity of light. The constant α_γ_ is the linear regression coefficient for acute doses of γ-rays for the same endpoint. The parametric form of Equation (8) is similar to the Katz cellular track structure model^27^, however assuming an initial linear dose component for γ-rays for *m* > 1.

In Equations (5) and (6), we considered several approaches to model the radiation quality dependence of the β-term. First we considered fitting values for γ-rays and low LET particles independently (H and He) while assuming β = 0 for higher LET particles. In a second approach we considered a simultaneous fit for γ-rays, H and He particles. Lastly we considered a global fit to the value of β however using the adjustment:

9 $$\beta (Z,E)={\beta }_{\gamma }[1-P(Z,E)]$$

Equation (9) allows the influence of the dose-squared term to smoothly diminish with increasing ionization density or LET similar to the result found in the track structure model which can be described using either multi-target, multi-hit, or α-β models as described by Katz^28^. These different approaches led to modest changes in fits and we used Equation (9) in the results below.

### Cell Survival Function

Dimajo *et al*.^29^ performed *ex-vivo* analysis of the dose response for cell survival in the Harderian gland after X–ray irradiation using male CBA/Cne mice. In their report, cell survival was well fit using the multi-target model with extrapolation number, *m*_*S*_, and radio-sensitivity parameter D_0_ using

10 $$S(D)=[1-{(1-{e}^{-D/{D}_{0}})}^{{m}_{s}}]$$

However, because of the mouse strain differences and possible differences between male and female mice, we also considered allowing D_0_ and *m*_*S*_ to be fit to the dose response data for tumor prevalence. We note an earlier estimate^21^ of these data found *m*_*s*_ = 3 and D_0_ = 2.6 Gy.

Considering the track structure model for particles^27^, the cell survival function is given by:

11 $$S(Z,E,F)=[1-{(1-{e}^{-D/{D}_{\gamma }})}^{{m}_{s}}]{e}^{(-{\sigma }_{0s}{P}_{s}(Z,E)F)}$$

where $${D}_{\gamma }=D[1-P{}_{S}(Z,E)]$$ and $${P}_{S}(Z,E)={[1-{e}^{(-\frac{{Z}^{\ast 2}}{{\kappa }_{S}{\beta }^{2}})}]}^{{m}_{s}}$$

### RBE Estimates

At low dose or fluence, RBE was calculated using the TE model, and is given by the ratio of linear induction coefficients. Here we used RBE_γ__Acute_, which compares low dose particle responses to acute γ-ray exposures using a linear fit of acute γ-ray responses doses near 1 Gy^1, 10^, with linear coefficient denoted as α_γ_. For the HG experiment the highest γ- ray dose of 7 Gy is not included in the RBE_γ__Acute_ estimate. This approach is used in the NASA quality factor (QF) model^1^ in order to be consistent with the linear response model used to represent the Atomic-bomb survivor solid cancer incidence data while reducing uncertainties related to low dose and dose-rate γ-ray responses.

The functional form of the RBE function at low dose where cell survival effects can be ignored are based on the linear or targeted effects assumption (TE), which uses a linear fit to acute γ-ray responses as the reference radiation is given by:

12 $$RB{E}_{TE}=[1-P(Z,E)]+\frac{6.24{{\rm{\Sigma }}}_{0}P(Z,E)}{{\alpha }_{\gamma }L}$$

We also considered RBE in the NTE model, however RBE estimates are dose dependent and do not limit to a constant value at low dose. Here it is convenient to define a cross-over dose where the contributions from the TE and NTE terms in the Hazard function of Equation (6) are equal:

13 $${D}_{cr}=\frac{\eta L}{6.24{\rm{\Sigma }}}$$

The RBE in the NTE model is a function of the particles absorbed dose, D and is given by^20^:

14 $$RB{E}_{NTE}=RB{E}_{TE}(1+\frac{{D}_{cr}}{D})$$

### Data Fitting

All statistical analysis and data fitting were done using STATA/SE version 14.1 (Stata Corp.). The value of TE or NTE model parameters are fitted across all particle beams using nonlinear least-squares data fitting weighted by the inverse of the variance. Tests of goodness of fit comparing the models described were made with the Akaike Information Criteria (AIC), Bayesian Information Criteria (BIC), and Adjusted R^2^ tests, which take into account differences in the number of parameters in the models considered. In the AIC and BIC tests, the model with the lowest test statistic provides the optimal fit, and in the Adjusted R^2^ test, the model with the highest values of the test statistic provides the optimal fit to the data.

### GCR Exposures

GCR exposures include primary and secondary H, He and HZE particles, and secondary neutrons, mesons, electrons, and γ-rays over a wide energy range. We used the HZE particle transport computer code (HZETRN) with quantum fragmentation model nuclear interaction cross sections^30, 31^ and Badhwar–O’Neill GCR environmental model to estimate particle energy spectra, φ_j_(Z,E) as described previously^1, 9^. These methods agree with spaceflight data in low Earth orbit, in transit to Mars and on the Mars surface to within 15% for dose and dose equivalent^9, 30, 32^.

For GCR, which are low dose and dose-rate exposures, the contributions of cell survival and the β term for tumor induction are assumed to be negligible, and the Hazard function reduces to the linear induction and NTE terms. We considered 1-year GCR exposures; however ignore possible protraction effects that could occur in a mouse, which is outside the scope of this paper. The main assumption we make is that the exposures are low dose and dose-rate which would be true for the annual GCR environment (~0.2 Gy/y) delivered over time periods of a few days or longer times.

For the TE model, a mixed-field pseudo-action cross section is formed by weighting the particle spectra, φ_j_(E) for particle species, *j*, contributing to GCR exposure evaluated with the HZETRN code with the pseudo-biological action cross section for mono-energetic particles and summing:

15 $${({{\rm{\Sigma }}}_{mixed}F)}_{TE}=\sum _{j}\int dE\,{\varphi }_{j}(Z,E){\rm{\Sigma }}({Z}_{j},E)$$

For the LET dependent model a similar expression is obtained using the functions described previously^14^.

For estimates of NTEs to GCR exposures Equation (15) is modified to include the last term in Equation (6), with the low fluence correction of Equation (7) playing an important role in predictions. Here we assume: (1) The probability that a bystander cell receives an oncogenic signal only occurs if the fluence is sufficiently high such that a nearby cell is traversed. (2) The time dependence of the bystander signals is a few days or less such that interactions of bystander signals from different HZE particles can be ignored. (3) The probability that a bystander cell is transformed by a direct hit at a different time is small and can be ignored. The effective mixed-field pseudo-action cross section in the NTE model then becomes

16 $${({{\rm{\Sigma }}}_{mixed}F)}_{NTE}=\sum _{j}\int dE\,{\varphi }_{j}(Z,E){\rm{\Sigma }}({Z}_{j},E)+{\eta }_{0}{L}_{j}(E){e}^{-{\eta }_{1}{L}_{j}(E)}(1-{e}^{-{N}_{Bys}({\varphi }_{j}(E))})$$

Because *N*_*Bys*_, the maximum area limiting bystander effects for particle *j*, has not been estimated in the Harderian gland experiments, we used estimates based on cell culture experiments^24–26^ of 4x, 10x, and 20x the cross-sectional area of the target cell nucleus in our results.

## Results

In Equation (8) the parameter *m* is the slope power describing the rise of the biological action cross section with increasing ionization density. Supplementary Table S1 shows results of fit for values of the parameter, *m*, of 2, 3, or 4, or fit with *m* as a free parameter. These results show the value *m* = 3 provided the best fit based on several statistical tests (Adjusted R^2^, AIC and BIC) and was then used in subsequent analysis, which indicates large values of biological effectiveness will occur for heavy ions and other high LET radiation types. Table 1 shows values of fitted parameters in the TE and NTE models described in the Methods section. The linear dose response terms for the particle data were similar in the models considered, however the NTE provided the optimal fits based on the AIC, BIC, and Adjusted R^2^ tests, which are statistical tests that account for the differences in the number of parameters used in the comparison of models (TE vs. NTE).

**Table 1:** Parameter estimates for combined ion data sets for targeted effects (TE) and non-targeted effects (NTE) models for the dose response for fraction tumor prevalence.

Parameter	TE	NTE
P_0_	0.0290 ± 0.0035 (<10^−4^)	0.0268 ± 0.0035 (<10^−4^)
σ_0,_ (μm^2^)	85.63 ± 17.13 (<10^−4^)	66.69 ± 40.68 (<10^−4^)
κ	713 ± 121 (<10^−4^)	750 ± 360 (<10^−4^)
σ_0S,_ (μm^2^)	22.65 ± 20.38 (0.100)	16.44 ± 52.62 (0.389)
κ_S_	469 ± 516 (0.405)	598 ± 1960 (0.761)
η_0,_ (keV/μm)^−1^	—	0.00048 ± 0.00033 (<10^−4^)
η_1,_ (keV/μm)^−1^	—	0.00281 ± 0.00320 (0.392)
Statistical Tests		
Adjusted R^2^	0.9999	0.9999
AIC	−260.00	**−264.65**
BIC	−249.79	**−250.35**

Parameter fits and standard deviations (including p-values) are described using survival function parameters m_s_ = 3 and D_0_ = 2.6 Gy, and tumor induction parameter m = 3. The model providing the optimal fit (lowest AIC or BIC values) is shown in bold-face.

**Figure 1:**
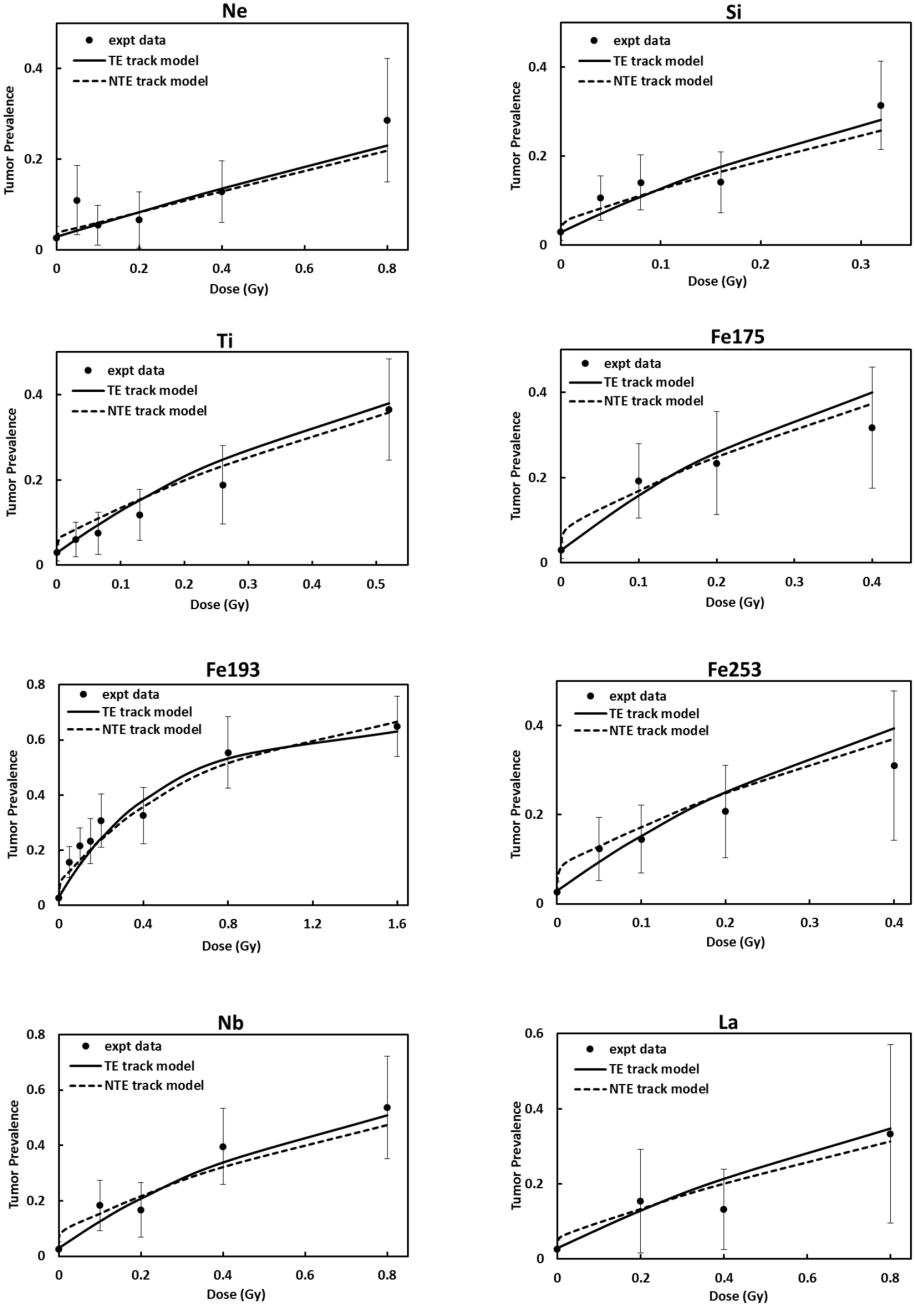
Comparison of TE and NTE models for Ne, Si, Ti, Fe, Nb and La beams to the experiments of Alpen *et al*.^12, 13^ and Chang *et al*.^14^, respectively for the dose response for Harderian gland tumor prevalence. Error bars represent standard errors in the experiments.

**Figure 2:**
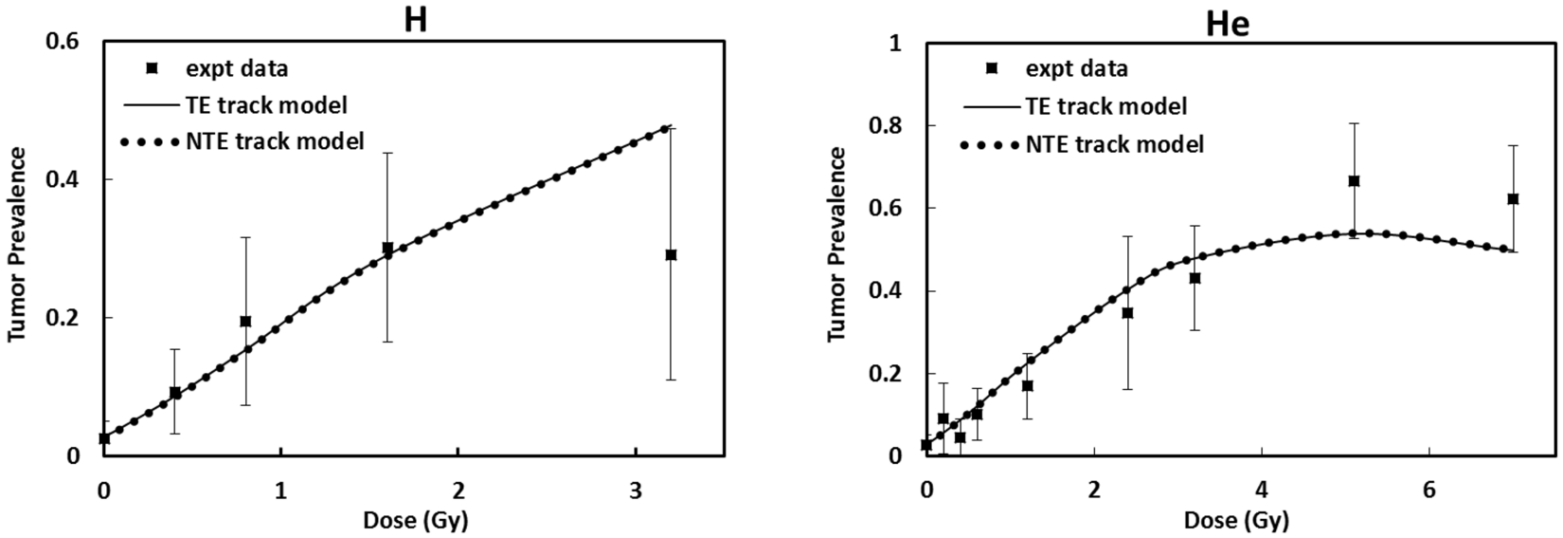
Comparison of targeted effects (TE) and non-targeted effects (NTE) models for proton and helium beams to the experiment of Alpen *et al*.^12^ for the dose response for Harderian gland tumor prevalence. Results show NTE are small for low LET particle beams.

Figure 1 shows results for tumor prevalence versus absorbed dose for the Ne (670 MeV/u), Si (260 MeV/u), Ti (1000 MeV/u), Fe (600 MeV/u), Fe (540 MeV/u), Fe (350 MeV/u), Nb (580 MeV/u) and La (593 MeV/u) beams, and the global fits in the TE and NTE models to these data. Fits to each beam individually slightly improve the results; however global fits are advantageous to consider for applications in GCR risk assessments. The results of Fig. 1 show that as LET increased the NTE predicts much larger TP at the lower doses (<0.1 Gy) compared to the TE model. In Fig. 2 the TE and NTE track structure dose response models for proton and helium beams are compared to the experimental data of Alpen *et al*.^12^. For these low LET particles the models fit well, while results for the TE and NTE models are nearly identical, suggesting NTE play a negligible role for low LET particles. The experiments for helium clearly show the reduction in TP at the highest doses, which is assumed to be due to cell killing in our model.

**Figure 3:**
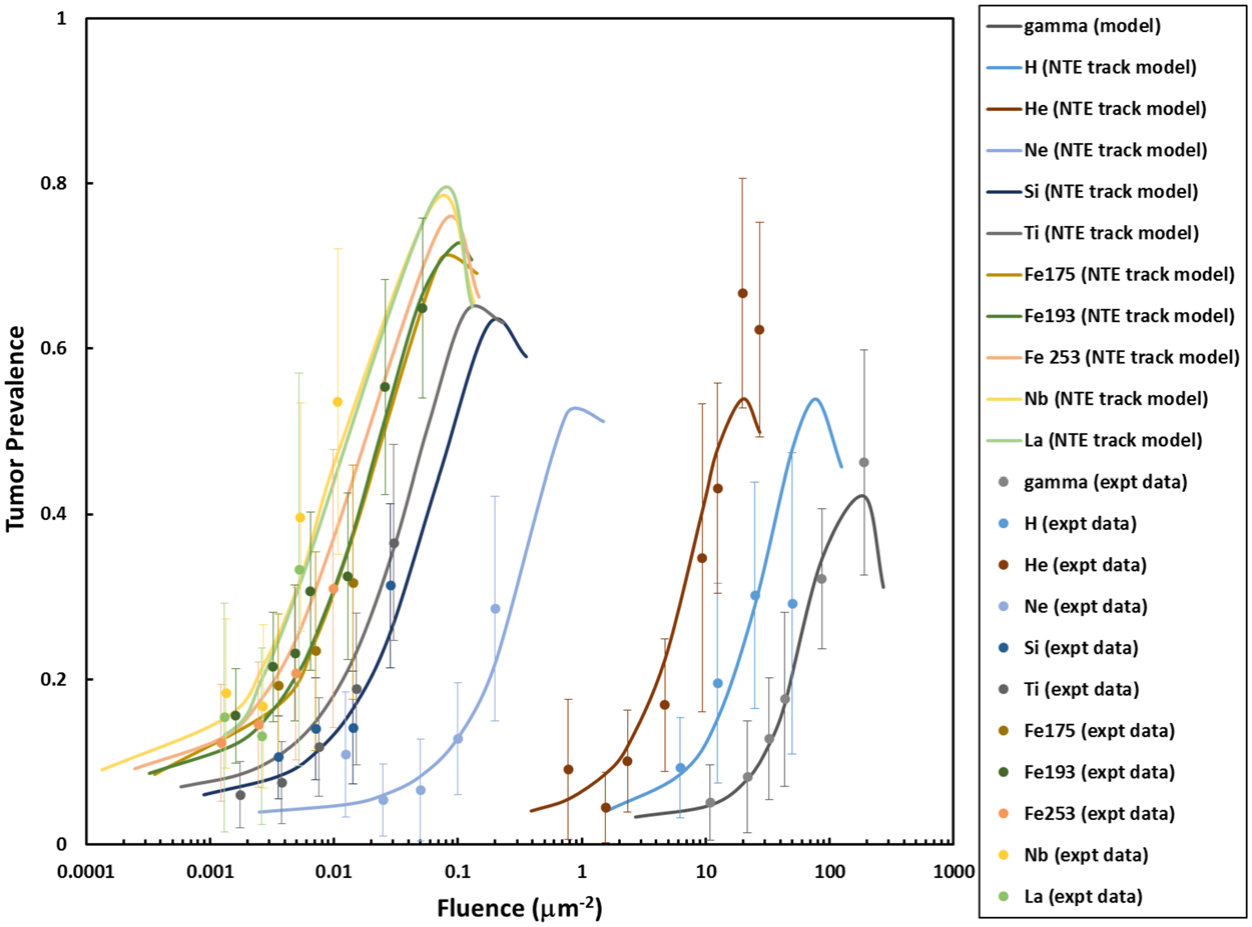
Comparison of the non-targeted effects (NTE) model of the percent Harderian gland tumor prevalence versus particle fluence to experiments of Alpen *et al*.^12, 13^ and Chang *et al*.^14^. Error bars represent standard errors in the experiments.

Figure 3 shows the combined data from various experiments^12–14^ for tumor prevalence versus the fluence of particles in the NTE model. Excellent agreement is found between the NTE model and the experiments. These results indicate that exposure to much less than one particle per cell nuclei in the Harderian gland increases TP by several-fold above the background TP for ^56^Fe and other heavy ions. Table 2 provides our predictions of dose-dependent RBEs in the NTE model with values at 0.1 and 0.01 Gy and the limiting value at higher dose shown. The RBE for heavy ions is predicted to increase as dose is decreased as described by Equation (14). This is due to the weak or non-existent NTE expected for γ-rays such that the nearly dose-independent NTE for heavy ions is being compared to a diminishing effectiveness for γ-rays at lowe doses. At higher doses the RBE decreases and TE’s dominate the heavy ion response. The RBE estimates in Table 2 are consistent with those found for other high-LET radiations, including ^56^Fe particles and fission neutrons, however the present results are unique in that the RBE’s for a wide range of particles are described that span the types of particles found in space. RBE estimates in the NTE model greatly exceed those of the TE model reaching a value of 50 relative to acute γ-ray exposures at 0.001 Gy for the most effective particle type studied.

**Table 2:** Estimates of Relative Biological Effectiveness (RBE) at different doses, cross-over dose, Dcr, defined as where the NTE and TE terms in Equation (12) are estimated to be equal.

*Z*	LET, keV/µm	D_cr_, Gy	RBE(NTE) TE limit at higher dose (>0.2 Gy)	RBE(NTE) at 0.1 Gy	RBE(NTE) at 0.01 Gy
1	0.4	0.0014	1.05 ± 0.02	1.07	1.20
2	1.6	0.0056	1.23 ± 0.07	1.30	1.91
10	25	0.0470	3.30 ± 0.63	4.86	18.83
14	70	0.0333	7.81 ± 1.88	10.41	33.80
22	107	0.0455	7.23 ± 1.73	10.52	40.15
26	175	0.0452	8.78 ± 2.20	12.75	48.48
26	193	0.0519	8.02 ± 2.00	12.19	49.67
26	253	0.0551	8.31 ± 2.11	12.89	54.12
41	464	0.0719	7.07 ± 1.86	12.15	57.91
57	953	0.0715	4.08 ± 1.10	7.00	33.29

The RBE(NTE) is relative to a linear response at higher doses (~1 Gy) of acute γ-rays.

**Figure 4:**
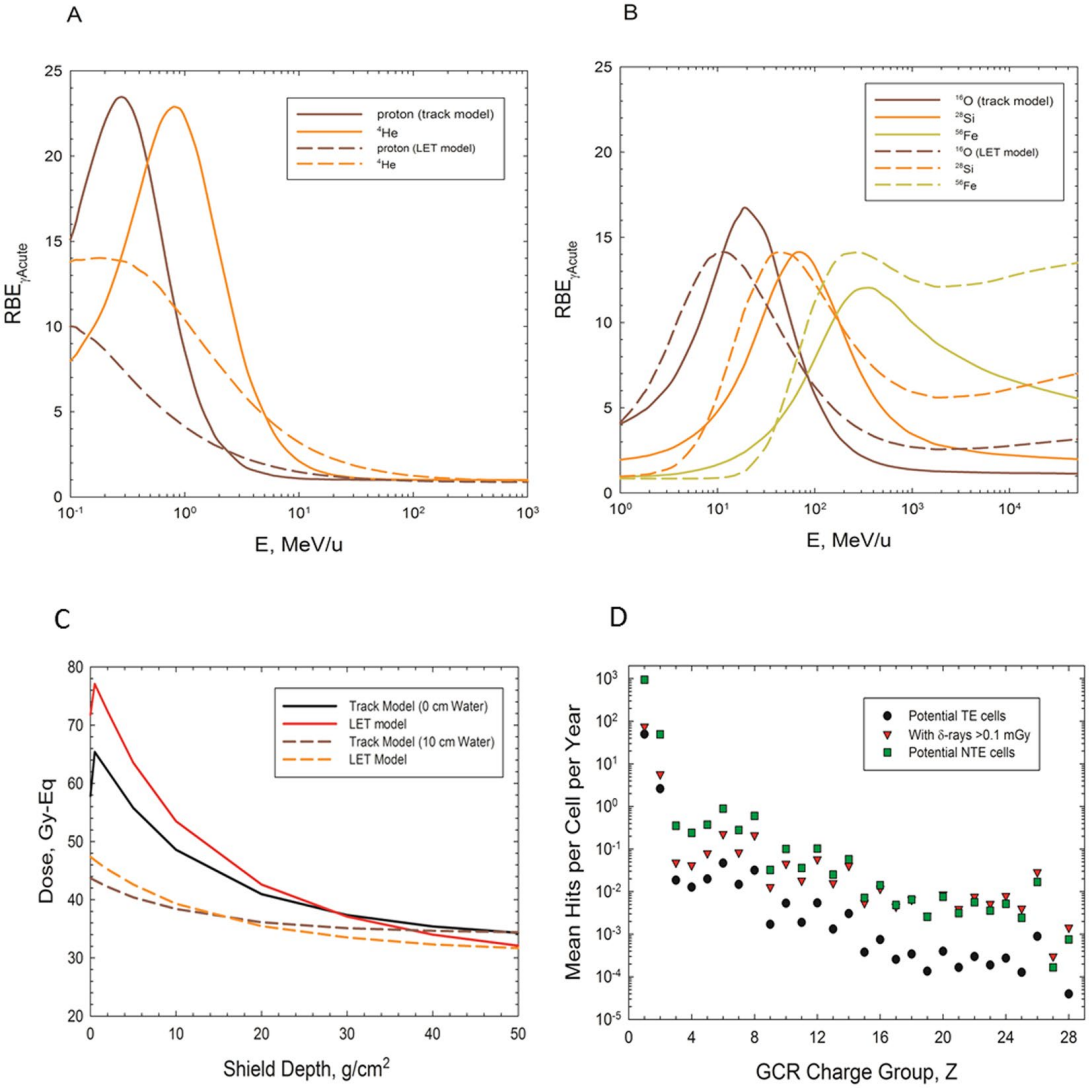
Comparison of relative biological effectiveness (RBE) factors for different particle types versus kinetic energy for the track structure and LET models and the mean number of cells at risk in annual GCR exposures. (**A**) Results for protons and ^4^He. (**B**) results for ^16^O, ^28^Si, and ^56^Fe. (**C**) shows a comparison of dose equivalent based on the RBE for Harderian gland tumor prevalence for the track structure and LET dependent targeted effects (TE) models versus aluminum shielding areal density for 0 and 10 cm of tissue depth. (**D**) Predictions of potential number of cells per year susceptible to targeted effects (TE) and non-targeted effects (NTE) for galactic cosmic ray (GCR) exposures at solar minimum assuming 20 g/cm^2^ aluminum shielding and 5 cm tissue depth. Also shown are mean number of cells hits per year by δ-rays with an absorbed dose >0.1 mGy. Calculations use the target area for epithelial cells in the Harderian gland of A_cell_ = 24 μm^2^ with NTE estimates using A_bys_ = 20 A_cell_ as described in the text.

Using the fits based on the TE models, Fig. 4 show the RBE_γAcute_ in the track structure model and LET model versus kinetic energy with Fig. 4A (proton and ^4^He) and Fig. 4B (^16^O, ^28^Si, and ^56^Fe). We note that the results of Fig. 4 used model parameters fit with the TE models, which leads to different RBE predictions compared to parameters derived from fits of the NTE model at higher doses in Table 2 where the first term in Equation (6) dominates. Clearly there are large differences that occur in the two approaches to extrapolate a limited number of experimental observations to the diversity of particles that appear in the GCR. Figure 4C compares predictions for different shielding amounts for the track structure and LET dependent TE models. The LET dependent model provides a higher TP prediction compared to the track structure dependent model at shallow to intermediate shielding depths (<20 g/cm^2^), while the track structure model shows higher TP at large shielding depths (>20 g/cm^2^).

The number of cells at risk include cells directly traversed by ions and bystander cells. Cells not directly traversed by GCR particles also receive small absorbed doses from δ-rays (<10 mGy) and the question arises on the importance of their contribution to tumor induction. In an earlier report a method to estimate the number of cells receiving δ-ray tracks alone above a dose threshold was formulated using radial dose models of energy deposition by ions^23^. In Fig. 4D predictions of potential number of cells per year susceptible to TE and NTE for annual GCR exposures at solar minimum assuming 20 g/cm^2^ aluminum shielding and 5 cm tissue depth are shown assuming A_Bys_ = 20 A_cell_. The mean number of cells hits per year by δ-rays with an absorbed dose >0.1 mGy are also shown for different GCR charge groups. The results of Fig. 4D show that cells potentially transformed by either TE or NTE from heavy ions are unlikely to be traversed by more than one heavy ion on an annual basis, which is an assumption made by the model of Equation (16). More frequent traversals by low LET protons and helium ions are predicted; however the probability of NTE transformation by these particles is small as shown by Fig. 2. Low energy protons and helium ions are high LET irradiation and will have large TE and NTE probabilities as predicted by Equations (8) and (7), respectively, however make-up only a small percentage (<10%) of the mean number of proton and helium hits in Fig. 4D. The number of cells traversed by δ-rays alone is similar to the number of NTE potential cells for heavy ions, while the number of NTE potential cells is much larger for low charge number, Z ions. However the biological effects of δ-rays will be similar to that of γ-rays. Using the γ-ray dose response data for Harderian gland tumors^11–14^ suggests the low absorbed doses to cells traversed by δ-rays leads to a small carcinogenic risk. The probability of NTE tumor induction estimated by fits to the Harderian gland experiments using Equation (7) is considered next.

**Figure 5:**
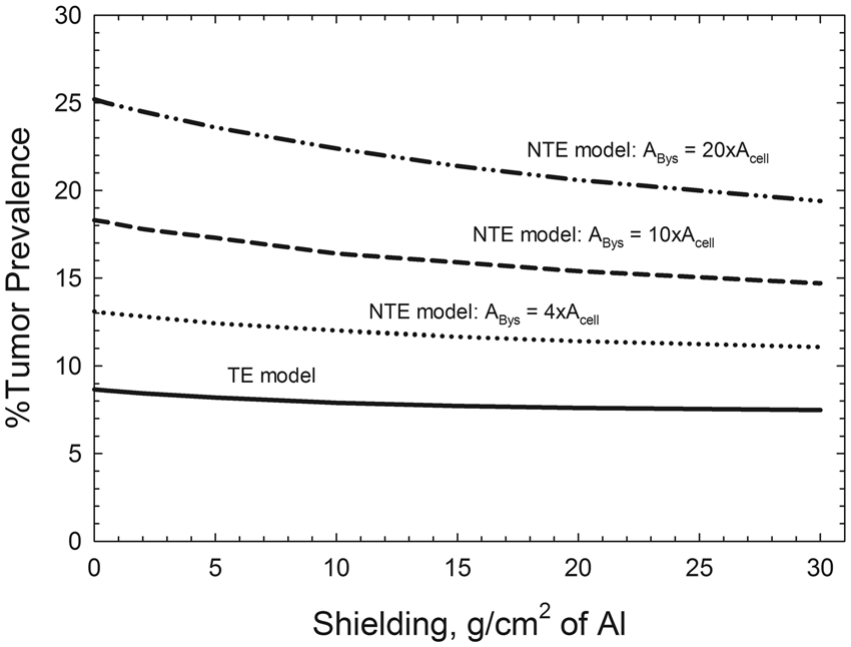
Predictions of % tumor prevalence for 1-year galactic cosmic ray (GCR) exposures at solar minimum versus aluminum shielding depth in targeted effects (TE) and non-targeted effects (NTE) model. Calculations assume 5 cm of tissue equivalent shielding and explore NTE predictions for several assumptions on the area encompassed by bystander cells susceptible to oncogenic signals from directly traversed cells.

In Fig. 5 we show the main result of this paper where %-TP for the TE and NTE models is compared. For the NTE model, predictions using different assumptions for the effective cross-sectional area for bystander cells that receive oncogenic signals from nearby directly traversed cells are shown. This result suggest that NTEs could dominate GCR cancer risks and thus represent the large uncertainty associated with determining low dose (<0.1 Gy) responses from HZE particles and other high LET radiation. The implications of this finding is further demonstrated in Table 3 which compares the resulting effective RBE estimates for GCR behind a typical spacecraft average shielding of 20 g/cm^2^ of aluminum to measurements of the GCR average quality factor (QF) from the Mars Science Laboratories transit from Earth to Mars^33^. Here the QF estimate is based on the LET dependent model of radiation quality^8^. The predictions of the NTE model suggest that a 2-fold enhancement in cancer risk would occur compared to the TE model predictions with further differences using LET as a descriptor compared to one based on descriptors of particle track structure.

**Table 3:** Predictions of galactic cosmic ray (GCR) averaged relative biological effectiveness (RBE) factors in targeted effect (TE) and non-targeted effects (NTE) models.

Model	GCR Averaged RBE
TE	5.0
NTE: A_Bys_ = 4 × A_nuc_	7.1
NTE: A_Bys_ = 10 × A_nuc_	8.0
NTE: A_Bys_ = 20 × A_nuc_	10.5
**MSL Data**	**GCR Averaged QF**
Mars Science Lab	3.8

Results are for 20 g/cm^2^ aluminum shielding with 5 cm tissue shielding. Also shown for comparison is the result of the measurement from the Earth to Mars transit phase by the Mars Science Laboratory (MSL) experiment^33^ for the GCR averaged quality factor (QF).

## Discussion

The NTE model is shown to predict a 2-fold or higher cancer risk compared to a TE model for chronic GCR exposures with predictions highly dependent on the number of bystander cells susceptible to oncogenic signals from cells directly traversed by heavy ions. The Harderian gland tumor induction studies^11–13^ has remained the most comprehensive data available to study the dose responses for a significant number of heavy ion types, including the recent studies with ^28^Si and ^48^Ti particles^14^. Nevertheless significant differences in the dose response and relative biological effectiveness factors (RBEs) have been reported between mouse studies for the Harderian gland with tissues that dominate human radiation cancer risk, including acute myeloid leukemia^34, 35^, hepatocellular carcinoma^34, 35^, mammary^36^ and colorectal tumors^37^. Older studies with fission neutrons show similar variations in RBEs for a wide-range of solid tumors^38–41^. These differences could limit the applicability of the predictions described in this paper based on the Harderian gland model. However both the National Council on Radiation Protection and Measurements (NCRP)^42^ and the International Commission on Radiological Protection (ICRP)^43^ have noted the Harderian gland experiments provide important information on radiation quality effects related to cancer risks. In addition, no other comprehensive data sets on low-dose cancer risks for HZE particles has been published, while studies with surrogate cancer endpoints in cell culture models are also sparse at the low doses appropriate to GCR exposure where the annual HZE particle dose in tissue is ~0.05 Gy/y near solar minimum, and less at other times in the solar cycle^1, 9, 31^.

Important evidence in support of NTEs as contributing to both cancer initiation and promotion is suggested by a large number of both *in-vitro* and *in-vivo* mechanistic studies^15–17, 44–46^, and studies with broad-beam irradiation such as alpha-particles^25, 26^ or HZE particles^19, 20^. The main limitation of the current NTE model predictions is the lack of an additional tumor type to understand the possible generality of our results. The Harderian gland tumor model enjoys a low background incidence of tumors, especially for the endpoint of TP at 600 days (~3%), which allows for low doses to be considered in experiments with a modest number of animals for each dose and particle type group. Other HZE particle tumor studies^34–37^ typically have higher background incidences (>10%) and have employed at most a single low dose (defined as doses where less than one particle traverses the target cell nucleus (~0.1 Gy)), while several low doses would be needed to understand the shape of the dose response and the relative contributions from TE and NTEs.

The effect of dose-rate in the present approach for low LET radiation follows from Equations (2) and (6) whereby for low dose-rate estimates the β-term is ignored. The values of α and β found for the Harderian gland experiment suggest a dose- and dose-rate reduction effectiveness factor (DDREF) of about 2.2 as described previously^10^. Grahn *et al*.^38^ performed chronic low dose-rate γ-ray exposures in B6CF1 male and female mice for 90 and 180-d duration, which were compared to acute γ-ray exposures. DDREF estimates for a variety of tumor types varied from about 1.7 to more than 5 in these experiments with an average value of about 2.7 found for the 90 day exposures^10^. Estimates of DDREF’s from the Atomic-bomb survivor cancer risk studies were about 1.3 in the BEIR VII report. However, Hoel^47^ suggests a DDREF between 2 and 3 using an improved analysis, which avoids the dose truncation model used by the BEIR VII committee^48^ and includes a cell sterilization term, which introduces downward curvature at higher doses. Thus the present results are similar to results of the Grahn *et al*.^38^ experiments and the analysis of Hoel^47^. In our previous publication^49^ we suggested that the DDREF to be used for δ-rays would be similar to that of γ-rays. For high LET radiation dose-rate modifiers are expected to a smaller compared to low LET radiation. Studies that have compared single acute exposure to dose-fractionation over several days with HZE particles have not reported important differences, especially at low dose^13, 14, 35, 50^, which suggests a DDREF near unity should be used for chronic high LET irradiation.

The major conclusion of this report is to recommend a priority be given to obtaining additional experimental data with a sufficient number of particle types with experiments performed with several low doses in the tissues predicted to dominate human radiation cancer risk, which are lung, stomach, liver, breast, and colon. Other studies are needed to understand the lethality of HZE particle and other high LET radiation induced tumors and possible reduced tumor latency compared to low LET and background tumors^1, 10, 35, 38, 50^.

## Electronic supplementary material

Supplementary File
