# Supplementary material for: Non-Targeted Effects Models Predict Significantly Higher Mars Mission Cancer Risk than Targeted Effects Models
Source: Sci Rep. 2017 May 12;7:1832. doi: 10.1038/s41598-017-02087-3 (PMC5431989; doi:10.1038/s41598-017-02087-3)
Supplement: Supplementary file 1 — Supplementary File [file 41598_2017_2087_MOESM1_ESM.pdf]

Supplementary File for:

Non-Targeted Effects Models Predict Significantly Higher Mars Mission Cancer Risk than Targeted Effects Models

Francis A. Cucinotta[a] & Eliedonna Cacao[a]
[a]University of Nevada Las Vegas, Las Vegas , NV, 89195, U.S.A

Supplementary Table S.1. Parameter estimates for γ-ray model and linear model of combined ion data for dose response for fraction tumor prevalence. Parameter fits and standard deviations (including p-values) for fixed values of *m* or allowing the value of *m* to be used as a fitting parameter are described using survival function parameters $m_s = 3$ and $D_0 = 2.6$ Gy. The model providing the optimal fit (lowest AIC or BIC values) is shown in bold-face.

Parameter	Fit with "m" free parameter	Fits with fixed "m " values		
**Fraction prevalence of Harderian Gland tumors after γ-ray irradiation**				
**m**	3.44 ± 2.83 (0.291)	2	**3**	4
**P_0**	0.0271 ± 0.0035 (0.008)	0.0344 ± 0.0265 (0.007)	**0.0412 ± 0.0239 (0.003)**	0.0450 ± 0.0234 (0.002)
**D_i (Gy)**	1.70 ± 1.02 (0.440)	2.61 ± 0.65 (0.013)	**1.88 ± 0.39 (0.028)**	1.55 ± 0.31 (0.070)
**Statistical Tests**				
**Adjusted R^2**	0.9994	0.9994	**0.9995**	0.9995
**AIC**	-18.25	-19.29	**-20.2**	-20.19
**BIC**	-18.41	-19.39	**-20.3**	-20.29
**Fraction prevalence of Harderian Gland tumors after particle irradiation**				
**m**	3.12 ± 0.57 ($<10^{-4}$)	2	3	4
**P_0**	0.0293 ± 0.0035 ($<10^{-4}$)	0.0282 ± 0.0039 ($<10^{-4}$)	**0.0290 ± 0.0035 ($<10^{-4}$)**	0.0299 ± 0.0036 ($<10^{-4}$)
**$\sigma_{0,}$ (μm^2)**	84.77 ± 17.80 ($<10^{-4}$)	97.51 ± 18.53 ($<10^{-4}$)	**85.63 ± 17.13 ($<10^{-4}$)**	79.84 ± 16.77 ($<10^{-4}$)
**κ**	692 ± 166 ($<10^{-4}$)	1176 ± 200 ($<10^{-4}$)	**713 ± 121 ($<10^{-4}$)**	556 ± 89 ($<10^{-4}$)
**$\sigma_{0S,}$ (μm^2)**	24.53 ± 24.53 (0.300)	13.34 ± 4.14 (0.020)	**22.65 ± 20.38 (0.100)**	27.11 ± 32.54 (0.370)
**κ_S**	484 ± 606 (0.428)	192 ± 78 (0.017)	**469 ± 516 (0.405)**	541 ± 688 (0.435)
**Statistical Tests**				
**Adjusted R^2**	0.9999	0.9999	**0.9999**	0.9999
**AIC**	-258.06	-247.88	**-260.00**	-258.52
**BIC**	-245.80	-237.67	**-249.79**	-248.30
